# Associations between Nighttime Traffic Noise and Sleep: The Finnish Public Sector Study

**DOI:** 10.1289/ehp.1205026

**Published:** 2012-10-01

**Authors:** Jaana I. Halonen, Jussi Vahtera, Stephen Stansfeld, Tarja Yli-Tuomi, Paula Salo, Jaana Pentti, Mika Kivimäki, Timo Lanki

**Affiliations:** 1 Finnish Institute of Occupational Health, Helsinki, Finland; 2 Department of Public Health, University of Turku and Turku University Hospital, Turku, Finland; 3 Centre for Psychiatry, Wolfson Institute of Preventive Medicine, Barts and The London School of Medicine and Dentistry, Queen Mary University of London, United Kingdom; 4 Department of Environmental Health, National Institute for Health and Welfare, Kuopio, Finland; 5 Department of Psychology, University of Turku, Turku, Finland; 6 Department of Epidemiology and Public Health, University College of London, London, United Kingdom

**Keywords:** cohort study, epidemiology, sleep disturbance, traffic noise

## Abstract

Background: Associations between traffic noise and sleep problems have been detected in experimental studies, but population-level evidence is scarce.

Objectives: We studied the relationship between the levels of nighttime traffic noise and sleep disturbances and identified vulnerable population groups.

Methods: Noise levels of nighttime–outdoor traffic were modeled based on the traffic intensities in the cities of Helsinki and Vantaa, Finland. In these cities, 7,019 public sector employees (81% women) responded to postal surveys on sleep and health. We linked modeled outdoor noise levels to the residences of the employees who responded to the postal survey. We used logistic regression models to estimate associations of noise levels with subjectively assessed duration of sleep and symptoms of insomnia (i.e., difficulties falling asleep, waking up frequently during the night, waking up too early in the morning, nonrestorative sleep). We also used stratified models to investigate the possibility of vulnerable subgroups.

Results: For the total study population, exposure to levels of nighttime–outside (L_night, outside_) traffic noise > 55 dB was associated with any insomnia symptom ≥ 2 nights per week [odds ratio (OR) = 1.32; 95% confidence interval (CI): 1.05, 1.65]. Among participants with higher trait anxiety scores, which we hypothesized were a proxy for noise sensitivity, the ORs for any insomnia symptom at exposures to L_night, outside_ traffic noises 50.1–55 dB and > 55 dB versus ≤ 45 dB were 1.34 (95% CI: 1.00, 1.80) and 1.61 (95% CI: 1.07, 2.42), respectively.

Conclusions: Nighttime traffic noise levels > 50 dB L_night, outside_ was associated with insomnia symptoms among persons with higher scores for trait anxiety. For the total study population, L_night, outside_ > 55 dB was positively associated with any symptoms.

Traffic noise is an environmental concern that is increasing with greater traffic volumes and urbanization (Moudon 2009; Pirrera et al. 2010). Traffic noise has been associated with cardiovascular outcomes (Sobotova et al. 2010; Sørensen et al. 2011), annoyance (Björk et al. 2006; Bluhm et al. 2004), and sleep disturbances (Basner et al. 2011; Brink 2011; Jakovljevic et al. 2006; Marks and Griefahn 2007; Stosic et al. 2009). The relatively high correlation between traffic noise and air pollution makes it difficult to estimate independent effects of noise on cardiovascular health (Foraster et al. 2011), but air pollution is less likely to confound associations between noise and sleep. Sleep is crucial for daytime performance because sleep disturbances may decrease cognitive functions (Cain et al. 2011) and increase the risk of absences from work (Salo et al. 2010). However, population-level evidence about the effects of nighttime traffic noise on sleep is very limited (Moudon 2009).

Existing studies on traffic noise and sleep problems, including difficulties initiating and maintaining sleep and tiredness in the morning, have been laboratory based (Babisch 2005; Öhrström et al. 2006). In the laboratory, noise exposure can be accurately defined and controlled, but the laboratory setting does not mirror the circumstances to which people are exposed in every-day life. In the laboratory studies, it is also difficult, if not impossible, to blind the participants from the study hypothesis, which may affect their responses. As a result, laboratory studies clearly suffer from limited generalizability to real-world settings (Basner et al. 2011; Eberhardt et al. 1987).

Studies that have examined the effects of traffic noise on vulnerable population groups also have been very limited, although it is known that some people experience noise more strongly than others [i.e., people who are noise sensitive (Stansfeld 1992)]. Noise sensitivity has somatic and psychological components and has been linked to hypertension, stress, depression, phobias, and hostility (Heinonen-Guzejev et al. 2004; Stansfeld 1992). It has also been correlated with annoyance and trait anxiety (Fyhri and Aasvang 2010; Stansfeld et al. 1993).

In this study, we combined sleep problems and other health-related data from the Finnish Public Sector Study cohort with data on modeled nighttime noise levels at the residential addresses of the cohort participants. Thus, we were able to study the association between levels of nighttime traffic noise with perceived sleep disturbances in a population-level setting in which participants were blinded to the aim of the study. Insomnia symptoms and duration of sleep were used as the outcomes. To investigate the possibility of vulnerable subgroups, we modeled interactions between noise and individual-level variables including higher trait anxiety scores, which may be a proxy marker of noise sensitivity, sex, marital status, occupational status, and obesity.

## Methods

*Study population.* The study population consisted of participants in the Finnish Public Sector Study cohort, an ongoing prospective study among employees working in 10 towns and 6 hospital districts (Kivimäki et al. 2010). These employees were from a wide range of occupational groups, from city mayors to semiskilled cleaners; the largest groups were nurses and teachers. The sex and age distribution of the cohort members is representative of the Finnish public sector employees (75% vs. 77% women, respectively; mean age 44 vs. 45 years). Surveys have been repeated every 4 years at the participating organizations for all employees starting from the year 2000. Surveys also were mailed twice (in 2005 and 2009) to participants who completed questionnaires while employed, but who subsequently left the participating organization. The ethics committees of the Finnish Institute of Occupational Health and the Hospital District of Helsinki and Uusimaa have approved the Finnish Public Sector Study, including this study. According to Finnish laws, written consent is not needed for research that uses survey and register data, as long as that participation is voluntary, and the respondents have been informed about the aims of the study and the possible data linkages (Finnish Ministry of Justice 1999). Moreover, the personal identification codes were not available to the researchers, and all analyses were performed anonymously using research identification codes.

We used survey responses from participants who resided in the cities of Helsinki and Vantaa (*n* = 7,089), because the modeled nighttime levels of traffic noise were available for these two cities. Addresses of the participants’ residences and the global positioning system (GPS) coordinates of these addresses were obtained from the Population Register Center (2000), which maintains the Population Information System in Finland. Survey responses from years 2004, 2005, 2008, and 2009 (on average, the response rate was 69%) were included, and for each participant, we chose the survey that included responses to all sleep questions and that was the closest to the noise modeling period (i.e., year 2006 in Helsinki and 2005 in Vantaa). Thus, the total number of participants in the analytic sample was 7,019.

*Dependent variables: sleep duration and insomnia symptoms*. Information on average duration of sleep and insomnia symptoms of each participant during the preceding 4 weeks was obtained from the surveys. Insomnia symptoms were measured with the Jenkins Sleep Problem Scale (Jenkins et al. 1988), which comprises questions on difficulty falling asleep, difficulty maintaining sleep during the night, waking up too early in the morning, and nonrestorative sleep (i.e., “Have you felt tired after normal sleep?”). Participants reported the frequency (never, 1 per month, 1 per week, 2–4 per week, 5–6 per week, and nearly every night) of each insomnia symptom. Each symptom was dichotomized as ≤ 1 night per week or ≥ 2 nights per week. In addition, we created a variable for “any insomnia symptom” based on the most frequent insomnia symptom the participant reported, which was also categorized as ≤ 1 night per week or ≥ 2 nights per week. Duration of sleep was reported in 30-min intervals and categorized into a “short sleep” variable (yes, ≤ 6.5 hr; no, ≥ 7 hr).

*Independent variables: nighttime traffic noise*. Outdoor levels of nighttime traffic noise (L_night, outside_ A-weighted night equivalent level, 2200–0700 hours) in the cities of Helsinki (Lahti et al. 2007) and Vantaa (Ramboll Finland Ltd. 2007) were modeled for major highways and for the main and collector streets using the Nordic prediction methods (Bendtsen 1999)—10-m grid size and a calculation height of 4 m, in accordance with the Environmental Noise Directive of the European Union (European Commission 2002). A collector street is a low-to-moderate-capacity street which functions as a feeder from local streets to a main street or highway. Temporal variation of traffic intensity was automatically measured by a traffic monitoring system at several main and collector streets in both cities. These data also were used to estimate nighttime traffic intensity for those streets that had information about 24-hr traffic intensity only. Smaller streets, which had no data on traffic intensity, were excluded from the noise model. First order reflection and the effect of noise barriers were included in the noise calculations. Data resolution was 0.1 dB, and no cut-off value was applied.

Noise levels at the most exposed façades of residential buildings were estimated using ArcMap 9.3.1 (ESRI, Redlands, CA, USA). First, all noise grid points located on top of the buildings were removed. Then, the two closest noise grid points were identified for each residence; the higher of these points was selected to represent the noise exposure. The GPS coordinates of the exposure points and the residences of the study participants were used for the linkage between the noise modeling and the survey data.

*Individual-level covariates*. We obtained questionnaire data on the following individual-level variables that may influence sleep: marital status (married or cohabiting vs. single); obesity (body mass index ≥ 30 vs. < 30 kg/m^2^); smoking status (current vs. no); moderate to heavy alcohol consumption (yes vs. no), defined as at least 24 drinks/week for men and 16 for women, corresponding to the levels of medium risk consumption established by the World Health Organization (WHO 2000); leisure time inactivity (yes vs. no), defined as < 2 metabolic equivalent task hours per day, which corresponds to approximately 30 min of brisk walking (Ainsworth et al. 2000); shift work (yes vs. no); and living with children < 6 years of age (yes vs. no). In addition, a trait anxiety score was derived from a 6-item Trait Anxiety Inventory, which is a short version of the 20-item Spielberger Trait Anxiety Inventory (Marteau and Bekker 1992; Spielberger et al. 1983), about how the person generally feels (“I feel calm,” I feel tense,” “I feel upset,” “I am relaxed,” “I feel satisfied,” “I am worried”). All items were rated on a 4-point scale: “Not at all” = 1, “A little” = 2, “To some degree” = 3, and “Very much so” = 4. When responses to at least four questions were available, we calculated the mean value of these points that was used as the score for trait anxiety (reverse scaling used for “I feel calm,” “I am relaxed,” and “I feel satisfied”). This value was then dichotomized as “higher score” (i.e., above the mean value ≥ 2.1) versus “lower score” and was used as a possible proxy indicator of noise-sensitive persons.

For each participant, we obtained information on age, sex, and occupational titles from the employers’ registers, which were used to classify individual socioeconomic status. Low socioeconomic status has been linked to short sleep duration (Stamatakis et al. 2007) and poor sleep quality (Patel et al. 2010). Using the *Classification of Occupations* (Statistics Finland 1987), as in our previous study (Salo et al. 2010), individuals were classified into three categories of occupational status based on their occupational title: high (upper-grade nonmanual worker), intermediate (lower-grade nonmanual worker), and low (manual workers). Because we had no information on the income of the participants, we used residence size (square meter) as another proxy for socioeconomic status (Black et al. 2004; Mannino et al. 2001) that was also classified into three categories: < 70, 70–100, > 100 m^2^. For each participant, information about size of their residences was obtained from the Population Register Center (2000).

Many chronic diseases may disrupt sleep (Agusti et al. 2011; Hsieh et al. 2011). Thus, a variable for “chronic disease” was derived using data on the presence of any baseline chronic condition (yes vs. no) obtained from the Drug Reimbursement Register kept by the Social Insurance Institution of Finland (KELA 2012). This register contains individual-level information on entitlements to special reimbursement for the cost of medication for chronic illnesses (hypertension, cardiac failure, ischemic heart disease, diabetes, asthma or other chronic obstructive lung disease, rheumatoid arthritis, and severe mental disorders), and the date when the special reimbursement was granted. In addition, information on cancers diagnosed in 2001 through 2005 was obtained from the Finnish Cancer Registry, which compiles all notifications of cancers nationwide (Teppo et al. 1994).

*Area-level covariates*. We used a grid database (Statistics Finland 2012) to obtain possible neighborhood-level socioeconomic confounders. These data are based on the total population in Finland, including information on the structure of the population, education, household income, and workplaces, by 250 × 250-m map squares that defined neighborhoods in this study. The GPS coordinates within each square and the coordinates of the residence of each participant were used for linking the data sets.

Living environments are likely to differ in traffic-noise levels by neighborhood socioeconomic disadvantage (i.e., high- and low-socioeconomic disadvantaged neighborhoods) (Havard et al. 2011), which may itself be associated with lowered sleep quality. Therefore, we built an index of socioeconomic disadvantage for each neighborhood using the grid database information on income (additive inverse of the log of median household income), education (percentage of adults > 18 years of age whose highest education level was elementary school), and unemployment rate (unemployed persons belonging to the workforce per the total workforce) as described earlier (Halonen et al. 2012), and used this index as a continuous variable. Population density (persons per square kilometer) was calculated by dividing the total number of residents in each square by the surface area of the square, and this calculation was used as a proxy for the degree of urbanization in the neighborhood. Missing data for the 250 × 250-m neighborhoods were replaced by the mean value of the variable in the eight surrounding neighborhoods.

*Statistical analyses*. Noise levels at the most exposed facades (L_night, outside_) were divided into four categories: ≤ 45 dB (*n* of participants = 4,399), 45.1–50 dB (*n* = 1,507), 50.1–55 dB (*n* = 716), and > 55 dB (*n* = 397). Associations between traffic noise and the dichotomized sleep variables were estimated using GENMOD logistic regression models (version 9.2; SAS Institute Inc., Cary, NC, USA). We ran three model specifications for all outcomes: model 1 included no adjustments; model 2 was adjusted for age (continuous), sex, socioeconomic status, and residence size; and model 3 was additionally adjusted for marital status, chronic disease, trait anxiety, as well as continuous variables for neighborhood socioeconomic disadvantage and population density. For the sensitivity analyses, we repeated model 3 excluding those who reported shift work (*n* = 801), including only those who were known to be employed at the time they responded to the survey (*n* = 5,623) because sleep is likely to improve after retirement (Vahtera et al. 2009), and adjusting for living with children < 6 years of age (yes or no; *n* = 5,366). In addition, we estimated the association between noise and any symptoms of insomnia ≥ 5 nights/week (*n* = 1,840) versus < 5 nights/week (Salo et al. 2010).

The possibility of vulnerable subgroups was explored by modeling multiplicative interactions between traffic noise (as a continuous variable) and individual-level covariates (age, sex, marital status, occupational status, residence size, obesity, smoking, physical inactivity, heavy alcohol use, trait anxiety, chronic disease, and children < 6 years) including only the product and main effect terms and no adjustments for covariates. If the *p*-value for the noise × covariate term was < 0.10, we estimated stratum-specific effect estimates for each category of the covariate.

## Results

Of the study population, 5,694 (81%) were women, and the mean ± SD age of the participants was 50.5 ± 11.2 years. Higher trait anxiety scores were equally common among men and women in this study population (42% in both groups). The mean ± SD external exposure level of nighttime traffic noise was 43 ± 7.1 dB. In Table 1, we present a more detailed description of the study population and the sleep outcomes by levels of nighttime traffic noise. The mean (± SD) population density in the lowest exposure group was 6,402 ± 5,972 persons/km^2^, and in the higher exposure groups it was 5,518 ± 5,029, 6,533 ± 5,673, and 8,413 ± 7,533.

**Table 1:** Descriptive statistics [n (%)] of the study participants and their neighborhoods by nighttime levels of traffic noise.

Covariate (no. of missing values)		Total	Nighttime traffic noise (dB)							*p*-Value^a^
			≤ 45	45.1–50	50.1–55	> 55				
Demographic variables
Sex	7,019	0.06
Men	1,325 (18.9)	860 (19.5)	262 (17.4)	129 (18.0)	74 (18.6)
Women	5,694 (81.1)	3,539 (80.5)	1,245 (82.6)	587 (82.0)	323 (81.4)
Married/cohabiting (88)	6,931	0.06
5,000 (71.2)	3,177 (73.0)	1,079 (72.6)	485 (69.2)	259 (66.8)
Obese (103)	6,842	0.09
1,057 (15.4)	646 (15.0)	237 (16.1)	108 (15.7)	66 (17.0)
Current smoker (103)	6,916	0.50
1,108 (16.0)	694 (16.0)	234 (15.7)	119 (17.0)	61 (15.7)
Physically inactive (62)	6,957	0.88
1,813 (26.1)	1,109 (25.4)	417 (27.8)	178 (25.2)	109 (27.8)
Moderate-to-heavy alcohol use (51)	6,968	0.22
	748 (10.7)	476 (10.9)	138 (9.2)	79 (11.1)	55 (14.0)
Trait anxiety score (204)	6,815	0.08
Higher score	2,870 (42.1)	1,836 (42.9)	612 (41.9)	276 (40.1)	146 (38.0)
Chronic disease	7,019	0.35
1,325 (18.9)	837 (19.0)	286 (19.0)	136 (19.0)	66 (16.6)
Children < 6 years of age (1,653)		5,366	0.28
		708	455 (13.5)	159 (13.8)	56 (10.4)	38 (12.9)
Occupational status (95)	4,858	0.77
High	2,892 (41.2)	1,797 (41.4)	614 (41.3)	287 (40.5)	194 (49.6)
Intermediate	3,094 (44.1)	1,963 (45.3)	662 (44.5)	324 (45.8)	145 (37.1)
Low	938 (13.4)	578 (13.3)	211 (14.2)	97 (13.7)	52 (13.3)
Residence size (22)	6,997	0.19
Large, > 100 m2	1,870 (26.7)	1,229 (28.0)	424 (28.3)	153 (21.5)	64 (16.1)
Intermediate, 70–100 m2	2,846 (40.7)	1,794 (40.9)	604 (40.2)	296 (41.6)	152 (38.3)
Small, < 70 m2	2,281 (32.6)	1,364 (31.1)	473 (31.5)	263 (36.9)	181 (45.6)
Sleep variables
Any symptom of insomnia (85)	6,961
3,546 (51.0)	2,228 (51.0)	743 (49.7)	359 (50.8)	216 (54.8)
Difficulties falling asleep (226)	6,793
	999 (14.7)	628 (14.7)	217 (14.9)	105 (15.3)	49 (12.6)
Frequently waking up during the night (116)	6,853
	2,696 (39.3)	1,692 (39.4)	573 (38.8)	278 (40.2)	153 (39.3)
Waking up too early in the morning (289)	6,730
	1,652 (24.5)	1,034 (24.6)	364 (25.2)	152 (22.2)	102 (26.3)
Nonrestorative sleep (168)	6,851
2,111 (30.8)	1,324 (30.9)	447 (30.3)	208 (30.0)	132 (33.9)
Short sleep (71)	6,948
1,568 (22.6)	981 (22.5)	340 (22.7)	165 (23.4)	82 (20.8)
aInteractions between noise and covariates in a model for any symptoms of insomnia.												

In the nonstratified regression analyses, we found some associations between traffic noise and sleep (Table 2). Compared with the ≤ 45 dB group, participants exposed to > 55 dB had odds ratios (ORs) of 1.32 [95% confidence interval (CI): 1.05, 1.65] for any insomnia symptom and 1.29 (95% CI: 1.01, 1.65) for nonrestorative sleep when adjusted for all of the individual- and neighborhood-level covariates in model 3. Results were similar when we excluded 801 shift workers or included only those with currently known employment status, and when we adjusted for living with children < 6 years old (data not shown). Association between levels of traffic noise > 55 dB and severe insomnia symptoms (≥ 5 nights/week) in the total study population (OR = 1.31; 95% CI: 1.02, 1.68) was similar to that for insomnia symptoms ≥ 2 nights/week.

**Table 2:** ORs and 95% CIs for insomnia symptoms and sleep duration by nighttime traffic noise in the crude and adjusted models.

Noise level (dB)	Any insomnia symptom	Difficulties falling asleep	Frequently waking up during the night	Waking up too early in the morning	Nonrestorative sleep	Short sleep duration (< 7 hr)
Model 1a
≤ 45 (reference)
45.1–50	0.95 (0.84, 1.07)	1.01 (0.86, 1.20)	0.98 (0.86, 1.10)	1.03 (0.90, 1.19)	0.97 (0.86, 1.11)	0.99 (0.86, 1.14)
50.1–55	0.99 (0.84, 1.16)	1.04 (0.83, 1.30)	1.03 (0.88, 1.22)	0.88 (0.72, 1.06)	0.96 (0.81, 1.14)	0.95 (0.79, 1.15)
> 55	1.16 (0.95, 1.46)	0.83 (0.61, 1.13)	1.00 (0.81, 1.23)	1.10(0.87, 1.39)	1.15 (0.92, 1.43)	1.11 (0.86, 1.43)
Model 2b
≤ 45 (reference)
45.1–50	0.95 (0.84, 1.07)	1.02 (0.86, 1.21)	0.98 (0.86, 1.11)	1.04 (0.90, 1.20)	0.98 (0.86, 1.12)	1.00 (0.86, 1.15)
50.1–55	0.97 (0.83, 1.14)	1.02 (0.81, 1.28)	1.02 (0.86, 1.20)	0.85 (0.69, 1.03)	0.96 (0.80, 1.14)	0.98 (0.81, 1.18)
> 55	1.15 (0.93, 1.42)	0.81 (0.59, 1.12)	1.01 (0.81, 1.25)	1.11 (0.87, 1.41)	1.15 (0.92, 1.44)	1.14 (0.88, 1.48)
Model 3c
≤ 45 (reference)
45.1–50	0.95 (0.84, 1.08)	1.06 (0.88, 1.27)	0.97 (0.85, 1.10)	1.04 (0.89, 1.21)	0.99 (0.86, 1.14)	0.98 (0.85, 1.14)
50.1–55	1.00 (0.84, 1.19)	1.05 (0.82, 1.34)	1.02 (0.85, 1.22)	0.85 (0.69, 1.05)	1.01 (0.84, 1.23)	0.99 (0.81, 1.22)
> 55	1.32 (1.05, 1.65)	0.83 (0.59, 1.17)	1.12 (0.88, 1.41)	1.24 (0.96, 1.61)	1.29 (1.01, 1.65)	1.12 (0.86, 1.47)
aCrude model. bModel adjusted for age, sex, occupational status, and residence size. cModel adjusted for age, sex, occupational status, residence size, marital status, chronic disease, trait anxiety, and neighborhood socioeconomic disadvantage and population density.												

**Figure 1:**
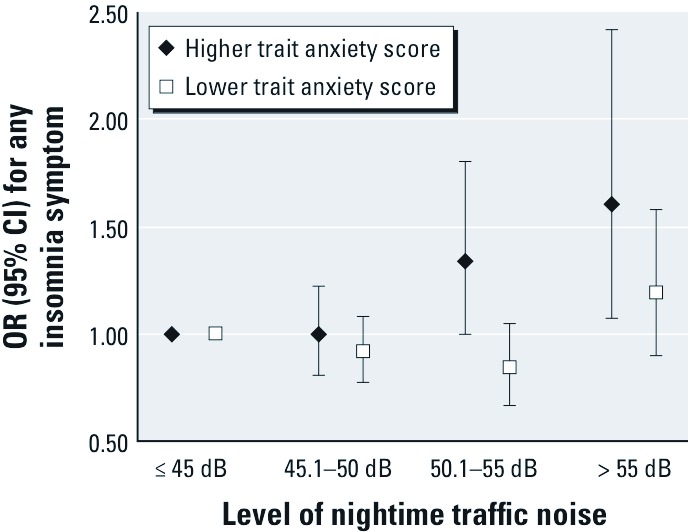
ORs (95% CIs) for any symptom of insomnia, L_night, outside_traffic noise among participants with higher trait anxiety scores and among those with lower trait anxiety score. Models adjusted for age, sex, occupational status, residence size, marital status, chronic disease, and neighborhood socioeconomic disadvantage and population density.

Interactions between covariates and noise were borderline significant (0.05 < *p* < 0.10) for sex, obesity, marital status, and trait anxiety, in the model for any insomnia symptom (Table 1). Stratified analyses indicated a stronger association between noise and any insomnia symptom among those with higher trait anxiety scores than among those with lower scores at 50.1–55 dB (OR = 1.34; 95% CI: 1.00, 1.80 compared with OR = 0.84; 95% CI: 0.67, 1.05) and at noise levels > 55 dB (OR = 1.61; 95% CI: 1.07, 2.42 compared with OR = 1.19; 95% CI: 0.90, 1.58) (Figure 1). Waking up frequently during the night seemed to be driving this finding [OR = 1.61; 95% CI: 1.13, 2.30 (higher score) compared with OR = 0.87; 95% CI: 0.58, 1.31 (lower score), at > 55 dB] (Table 3). Associations between noise exposure > 55 dB and any insomnia symptoms were stronger among women than among men and among obese compared with nonobese participants (Table 3).

**Table 3:** ORs and 95% CIs for any symptom of insomnia and individual sleep disorders by trait anxiety, sex, marital status, and obesity.

Nighttime traffic noise (dB)	Any insomnia symptom	Difficulties falling asleep	Frequently waking up during the night	Waking up too early in the morning	Nonrestorative sleep
Higher trait anxiety score
≤ 45 (reference)
45.1–50	0.99 (0.81, 1.22)	1.19 (0.95, 1.49)	1.09 (0.89, 1.32)	1.00 (0.82, 1.22)	0.91 (0.75, 1.10)
50.1–55	1.34 (1.00, 1.80)	1.27 (0.94, 1.72)	1.25 (0.96, 1.64)	0.98 (0.74, 1.29)	1.24 (0.95, 1.62)
> 55	1.61 (1.07, 2.42)	0.79 (0.51, 1.24)	1.39 (0.97, 1.99)	1.61 (1.13, 2.30)	1.33 (0.93, 1.89)
Lower trait anxiety score
≤ 45 (reference)
45.1–50	0.91 (0.77, 1.08)	0.83 (0.60, 1.13)	0.87 (0.73, 1.04)	1.07 (0.85, 1.34)	1.10 (0.89, 1.35)
50.1–55	0.84 (0.67, 1.05)	0.72 (0.46, 1.12)	0.86 (0.67, 1.10)	0.69 (0.49, 0.98)	0.81 (0.59, 1.11)
> 55	1.19 (0.90, 1.58)	0.86 (0.50, 1.47)	0.95 (0.69, 1.30)	0.87 (0.58, 1.31)	1.26 (0.89, 1.78)
Mena
≤ 45 (reference)
45.1–50	1.05 (0.77, 1.43)	0.71 (0.44, 1.14)	1.07 (0.78, 1.48)	0.84 (0.57, 1.24)	1.13 (0.80, 1.59)
50.1–55	0.76 (0.50, 1.16)	0.77 (0.41, 1.46)	0.76 (0.48, 1.19)	0.86 (0.51, 1.45)	0.92 (0.57, 1.49)
> 55	0.92 (0.54, 1.57)	1.02 (0.35, 1.89)	0.93 (0.53, 1.65)	1.07 (0.57, 2.02)	0.68 (0.36, 1.31)
Womena
≤ 45 (reference)
45.1–50	0.92 (0.80, 1.07)	1.13 (0.93, 1.37)	0.95 (0.82, 1.09)	1.08 (0.92, 1.27)	0.96 (0.82, 1.13)
50.1–55	1.06 (0.88, 1.29)	1.11 (0.85, 1.45)	1.08 (0.89, 1.32)	0.85 (0.67, 1.07)	1.06 (0.85, 1.31)
> 55	1.43 (1.11, 1.85)	0.83 (0.57, 1.21)	1.16 (0.90, 1.51)	1.28 (0.96, 1.71)	1.46 (1.11, 1.91)
Living alonea
≤ 45 (reference)
45.1–50	1.02 (0.79, 1.32)	1.10 (0.79, 1.52)	1.09 (0.85, 1.41)	1.15 (0.86, 1.53)	1.14 (0.87, 1.50)
50.1–55	1.08 (0.78, 1.51)	1.20 (0.79, 1.80)	1.10 (0.78, 1.53)	0.77 (0.52, 1.14)	1.04 (0.72, 1.48)
> 55	1.42 (0.95, 2.15)	1.02 (0.59, 1.77)	1.15 (1.01, 2.27)	1.21 (0.77, 1.91)	1.20(0.78, 1.86)
Married/cohabitinga
≤ 45 (reference)
45.1–50	0.92 (0.79, 1.07)	1.01 (0.81, 1.27)	0.92 (0.79, 1.08)	0.99 (0.83, 1.19)	0.93 (0.79, 1.11)
50.1–55	0.93 (0.75, 1.25)	0.95 (0.69, 1.31)	1.00 (0.81, 1.25)	0.88 (0.68, 1.13)	1.00 (0.79, 1.27)
> 55	1.27 (0.96, 1.68)	0.76 (0.48, 1.20)	0.98 (0.73, 1.31)	1.25 (0.91, 1.72)	1.30 (0.96, 1.77)
Not obesea
≤ 45 (reference)
45.1–50	0.93 (0.81, 1.08)	1.01 (0.82, 1.24)	0.98 (0.85, 1.14)	1.02 (0.86, 1.21)	0.97 (0.82, 1.13)
50.1–55	0.96 (0.79, 1.16)	1.10 (0.83, 1.45)	1.07 (0.87, 1.30)	0.84 (0.75, 1.19)	1.00 (0.80, 1.25)
> 55	1.23 (0.94, 1.56)	0.72 (0.48, 10.9)	1.14 (0.87, 1.48)	1.22 (0.91, 1.64)	1.29 (0.97, 1.70)
Obesea
≤ 45 (reference)
45.1–50	0.99 (0.71, 1.38)	1.18 (0.77, 1.78)	0.88 (0.64, 1.22)	1.22 (0.85, 1.74)	1.08 (0.77, 1.53)
50.1–55	1.05 (0.67, 1.66)	0.79 (0.43, 1.45)	0.85 (0.54, 1.33)	0.57 (0.33, 0.99)	1.04 (0.65, 1.65)
> 55	2.08 (1.14, 3.81)	1.44 (0.74, 2.82)	1.09 (0.62, 1.91)	1.38 (0.77, 2.48)	1.18 (0.66, 2.12)
aModels adjusted for age, sex, occupational status, residence size, marital status, chronic disease, trait anxiety, and neighborhood socioeconomic disadvantage and population density.										

## Discussion

We studied the association between exposure to nighttime traffic noise and sleep in a population-level setting where the participants were blind to the aim of the study. We found positive associations between nighttime noise levels > 50 dB and insomnia symptoms ≥ 2 nights per week among participants with higher trait anxiety scores. In the total study population, insomnia symptoms were associated with noise > 55 dB.

*Comparison with other studies*. Population studies of associations between traffic noise and sleep among vulnerable population groups are scarce (Basner et al. 2011). Thus, our finding of an association between noise and insomnia symptoms among those with higher trait anxiety scores is among the first published evidence that nighttime noise levels associated with sleep problems may be lower among vulnerable individuals than among persons in the general population. In a recent population study, Dratva et al. (2012) also reported that health effects of transportation noise may be greater among residents with a medical condition (e.g., diabetes, hypertension, or cardiovascular disease) than among other residents of an area with a mean level of nighttime traffic noise of 38.7 dB.

We also found an association between noise levels > 55 dB and any symptom of insomnia in the entire study population. This result is in line with findings reported from a study in Switzerland where sleep disturbances were predicted by nighttime traffic noise > 47 dB, but also by individual-level variables such as age, sex, socioeconomic status, body mass index, financial satisfaction, and satisfactions with personal relationships (Brink 2011). Noise levels > 65 dB have also been associated with sleep problems among women in Japanese cities (Kageyama et al. 1997). Although the estimated outdoor noise levels in our study were moderate, with the highest level of exposure comparable to the noise from loud conversation (~ 55 dB), indoor noise levels would have been further diminished because all residential buildings in Finland have good insulation and triple-glazed windows (minimum standard is double-glazed windows) against the harsh climate, which reduce the levels of traffic noise indoors (Garg et al. 2011). Thus, we would expect the association between traffic noise and sleep problems to be evident at lower levels of outdoor traffic noise in areas with less insulated buildings if the association is causal. Associations might also be stronger with measures of intermittent noise exposure (e.g., numbers of noise events or the maximum levels of noise during the night) (Eberhardt et al. 1987), but the frequency of noise events cannot be reliably modeled on a population level.

We found weak positive associations between the highest level of noise and frequently waking up too early in the morning and nonrestorative sleep and a weak negative association with difficulty falling asleep. Previously, in a study of 911 adults, participants living along a street where the nighttime noise level was > 45 dB were more likely to report difficulty falling asleep, being woken up, and tiredness after sleep than were those exposed to noise levels < 45 dB (Stosic et al. 2009). In another study, Öhrström et al. (2006) found a correlation between nighttime traffic noise and waking up during the night among adults. However, effect estimates for associations were not reported for either of these studies. Results from a laboratory study of adult participants indicated an association between exposure to nighttime traffic noise and disturbed sleep continuity. The results were based on self-reports and on an objective measure, but the researchers did not find an association with self-reported difficulties in falling asleep (Basner et al. 2011). However, laboratory and field studies are not directly comparable. For example, self-reported awakenings used in population studies may be associated with higher noise levels than objective electroencephalogram-defined measures of sleep disturbance used in laboratory studies (WHO 2009). Population-level associations between average levels of noise and sleep outcomes over time also represent longer-term effects of noise, which means that habituation to noise may play a more significant role than in laboratory settings (Basner et al. 2011). In addition, laboratory studies are often conducted in healthy, young subjects who may not be particularly sensitive to noise (Marks and Griefahn 2007).

*Implications*. Traffic noise > 50 dB was associated with any symptom of insomnia among those with higher trait anxiety scores; noise levels > 55 dB was associated with any symptom in the total study population. The current interim guideline for levels of outdoor nighttime traffic noise, as recommended by the WHO, is 55 dB (WHO 2009), which, based on our results, may not fully protect vulnerable population groups. On the other hand, the ideal WHO guideline for nighttime–outside noise levels of 40 dB may be too restrictive in areas where buildings are heavily insulated. Salo et al. (2010) found that disturbed sleep may reduce working capacity. Thus, factors that affect sleep quality may have adverse effects on daily activities as well as public health. Trait anxiety itself has been associated with poor sleep quality (Augner 2011), which may explain why persons with higher trait anxiety scores in our study population appeared to be vulnerable to insomnia symptoms at lower noise levels than levels associated with symptoms in the population as a whole.

Our results support actions towards limiting traffic noise exposure. These include the use of silent tires and noise-reducing road surfaces (Moudon 2009), reducing speed limits, and providing adequate sound barriers (Murphy and King 2011). Within cities, sound barriers often cannot be used, but noise insulation can be used in buildings. For example, triple-glazed windows not only save energy but also reduce environmental noise levels inside buildings (Garg et al. 2011).

*Limitations*. This study was subject to some limitations. The generalizability of our findings is limited by the fact that our study population consisted mainly of working female adults in an area with modest noise levels. Further, longitudinal population-level studies that are focused on other age groups are needed.

Another limitation is exposure misclassification caused by modeling procedures. The traffic intensities on the small streets were not available for the calculations in the noise modeling, which may have misclassified the exposure of participants who lived along these small streets. A further limitation is that we did not have data on the intensity of nighttime traffic for all streets when estimating average noise levels, although the available data on temporal variation were used for modeling. In addition, we could not account for variation in noise levels according to the location of bedrooms in relation to streets. Finally, some survey data were collected before the noise modeling was performed.

## Conclusions

In this study, we found an association between nighttime traffic noise > 50 dB and any symptom of insomnia among study participants with higher versus lower trait anxiety scores. In the total study population, insomnia symptoms were associated with nighttime traffic noise > 50 dB.
