# **Erratum: Halonen et al. “Associations between Nighttime Traffic Noise and Sleep: The Finnish Public Sector Study” **[120:1391–1396 (2012)]

**DOI:** 10.1289/ehp.121-a147

**Published:** 2013-05-01

In the article “Associations between Nighttime Traffic Noise and Sleep: The Finnish Public Sector Study,” by Halonen et al.”  [Environ Health Perspect 120:1391–1396 (2012)], the last sentence of the “Conclusions” was incorrect: The nighttime traffic noise level associated with insomnia symptoms in the total study population should have been “> 55 dB” instead of “> 50 dB.”

EHP regrets the error.

